# Using Living Labs to Explore Needs and Solutions for Older Adults With Dementia: Scoping Review

**DOI:** 10.2196/29031

**Published:** 2021-08-19

**Authors:** Henk Verloo, Adrien Lorette, Joëlle Rosselet Amoussou, Estelle Gillès de Pélichy, Alcina Matos Queirós, Armin von Gunten, Elodie Perruchoud

**Affiliations:** 1 School of Health Sciences HES-SO Valais and Department of Nursing Sciences University of Applied Sciences and Arts Western Switzerland Sion Switzerland; 2 Service of Old Age Psychiatry Lausanne University Hospital Prilly-Lausanne Switzerland; 3 Psychiatry Library, Education and Research Department Lausanne University Hospital and University of Lausanne Prilly-Lausanne Switzerland; 4 Service of Old Age Psychiatry Sector West, Prangins Hospital Lausanne University Hospital Prangins Switzerland; 5 Department of Health and Social Welfare Lausanne Switzerland; 6 Institute of Biomedical Sciences Abel Salazar University of Porto Porto Portugal; 7 Head of the Service of Old Age Psychiatry Lausanne University Hospital Prilly-Lausanne Switzerland

**Keywords:** living lab, aged, dementia, cognitive dysfunction, long-term care, primary health care, technology, mobile phone

## Abstract

**Background:**

Numerous living labs have established a new approach for studying the health, independent living, and well-being of older adults with dementia. Living labs interact with a broad set of stakeholders, including students, academic institutions, private companies, health care organizations, and patient representative bodies and even with other living labs. Hence, it is crucial to identify the types of cocreations that should be attempted and how they can be facilitated through living labs.

**Objective:**

This study aims to scope publications that examine all types of living lab activities, exploring the needs and expectations of older adults with dementia and seeking solutions, whether they live in the community or long-term health care facilities (LTHFs).

**Methods:**

This scoping review was reported according to the PRISMA (Preferred Reporting Items for Systematic Reviews and Meta-analyses) recommendations for the extension of scoping reviews. We searched six bibliographic databases for publications up to March 2020, and a forward-backward citation chasing was performed. Additional searches were conducted using Google Scholar. The quality of the selected papers was assessed.

**Results:**

Of the 5609 articles identified, we read 58 (1.03%) articles and retained 12 (0.21%) articles for inclusion and final analysis. All 12 articles presented an innovative product, developed in 4 main living labs, to assist older adults with cognitive disorders or dementia living in the community or LTHFs. The objectives of these studies were to optimize health, quality of life, independent living, home care, and safety of older adults with cognitive disorders or dementia, as well as to support professional and family caregivers or reduce their burdens. The overall methodological quality of the studies ranged from poor to moderate.

**Conclusions:**

This scoping review identified several living labs playing a pivotal role in research aimed at older adults with dementia living in the community or LTHFs. However, it also revealed that living labs should conduct more better-quality interventional research to prove the effectiveness of their technological products or service solutions.

**International Registered Report Identifier (IRRID):**

RR2-10.2147/SHTT.S233130

## Introduction

### Background

The world’s population of people aged >65 years is growing rapidly. In Europe, their proportion has increased from 14% in 2010 to 28% in 2020 [1]. According to the World Health Organization, approximately 20% of people aged ≥65 years have difficulties performing some of the activities of daily living (ADL) or instrumental ADL, often due to reduced mobility, weakened muscular strength, and disorders linked to cognitive disorders [2]. Innovative technologies or services are being used more frequently to provide responses to health problems, particularly for those affected by dementia [3]. In parallel, health care professionals and individual citizens want to participate in relevant, innovative, and implementable solutions that challenge the mainstream conceptions of the targets of health innovation [4]. Recent years have seen numerous studies reporting the advantages of adopting user-centered design approaches for developing innovative solutions. These approaches question users about their needs or observe their behavior with respect to a product, technology, or piece of equipment [5]. More recently, design research has evolved from a user-centered approach, wherein users are considered experimental subjects, to a more participatory approach, wherein users are considered partners [6]. This perspective points to the utility of design methods oriented toward increasing user and stakeholder participation, whether they are nonspecialists or professionals [7,8]. The emergence of living lab (LL) approaches has enabled researchers to go beyond the user-centered vision by adopting a *user-driven* perspective supported by other stakeholders [6]. LLs can turn the main beneficiary of a problem’s resolution into an actor with a key role in a scientific process [9].

There are many different definitions of an LL depending on the domain and the author’s research field; therefore, a widely recognized definition is lacking [10]. Depending on the definition, LLs are considered as a methodology for user-driven innovation; a user-driven, open-innovation ecosystem; a focus group involving users and stakeholders; or even an experiment in the environment [6]. This scoping review retained the definition presented by Bergvall-Kåreborn and Ståhlbröst [11]: “a living lab is a user-centric innovation environment built on every-day practice and research, with an approach that facilitates user influence in open and distributed innovation processes engaging all relevant partners in real-life contexts, aiming to create sustainable values.” With regard to older adults with dementia in different health care settings, Bergvall-Kåreborn and Ståhlbröst [11] also stated that an LL could be “a pragmatic research environment, which openly engages all relevant partners with an emphasis on improving the real-life care of people living with dementia through the use of economically viable and sustainable innovation” [12]. LLs can be viewed as settings for open innovation that provide collaborative platforms for research, development, and experimentation in real-life contexts using specific methodologies and tools [13]. Følstad [14] described nine characteristics of LLs, four of which are discovery, evaluation, familiar contexts, and a focus on the medium to long term. The other five contribute to the variety of LLs as they may or may not be displayed: the investigation of the context, active roles for the users, technical testing, real-world contexts, and multiple settings [14]. In the context of ever-increasing worldwide economic competition, it is becoming necessary for industries and companies to innovate incessantly. However, it has been estimated that 70% of the innovative products and services they develop cannot find a market because they do not meet the real-world user needs [15]. Given that LL solutions are developed under conditions that are designed to be closer to reality and that they can produce more effective solutions to the needs of end users, LLs represent a considerable advantage in many industrial and economic sectors [16]. By using LL platforms and methodologies, companies and health care institutions can reach beyond their own boundaries, follow an open-innovation model [17], and integrate outsiders into the cocreation of products [18], experiences, designs, quality implementation strategies, and service development [17]. LLs often act as intermediaries or innovation facilitators for the cocreation process by providing structure and governance [19,20]. The key components of LLs include information and communication technology (ICT), management, stakeholders, research, and methods of cocreation and product testing [12]. The ICT and infrastructure component reflects the role that new and existing ICT can play in facilitating new means of cooperating and cocreating innovations among stakeholders. The research symbolizes the collective learning and thinking that occurs in an LL and should contribute to both theory and practice. Technological research partners can also provide direct access to the panels of older adult testers of new products, which can benefit the development of technological innovation with regard to criteria such as ease of use [12].

### LLs for Older Adults With Dementia

Dementia is a progressive, disabling, chronic disease affecting 5% of all people aged >65 years and >40% of people aged >90 years [21]. Older adults with dementia need a great deal of support and assistance, and this need increases with the progression of the disease [22]. Nevertheless, most older adults prefer to live in their own homes for as long as possible, even if they risk falls, are disabled, or are physically and mentally impaired [23]. Although this decreases the pressure on nursing homes and other long-term health care facilities (LTHFs), it increases pressure on both informal family caregivers and community health professionals [24]. Some research and development has been conducted on cognitive prosthetic devices; however, there are few relevant tools, solutions, or technologies specifically for people with dementia [25].

To the best of our knowledge, there are no clear overviews of the research conducted by LLs either using modern assistive technology specifically designed for older adults with cognitive impairment or dementia or based on their observed and expressed needs. Numerous studies have addressed the areas of concern for aging populations in general rather than specifically for those with dementia [26]. Some studies have reported on the use of general memory aids that can be used by those affected by memory problems and other cognitive impairments [27]. These studies were often conducted in traditional laboratory settings and did not include older adults in their natural environments. Although laboratory studies are easier to control, their ecological validity is limited [28]. Considering the needs of older adults with dementia in conjunction with relevant technologies has led to the identification of potentially innovative solutions for cognitive reinforcement. The increasing drive to develop innovative, cost-effective dementia care strategies will only work effectively if innovative technologies meet the real needs of people living with dementia. These processes are often only discussed with their informal or professional caregivers, yet there is evidence that people with dementia are very capable of participating [29]. Involving them in the studies of their day-to-day life is challenging; however, because of their impaired cognitive abilities, studies that do not include them will face difficulty demonstrating the potential effects of implementation in real life [29]. LLs can involve people in their natural environments, thus providing more environmentally valid evaluations in the context of innovations for dementia [30].

The literature already contains attempts to explain and analyze the effects of LLs on technology and communication [31,32]. However, the many different and separate needs of older adults with dementia and their respective solutions remain underresearched [33]. This study aims to scope publications examining all the types of LL activities, exploring the needs and expectations of older adults with dementia, and suggesting solutions for them, whether they live in the community or in LTHFs. The following research question defined our search: “What does the literature say about living labs whose activities are dedicated to older adults with dementia living in the community or in LTHFs?” The overall outcomes of this scoping review will provide useful insights into existing activities and identify any remaining gaps in the services provided and the research conducted by LLs [34]. It will summarize knowledge on the contributions of (old age) LLs exploring needs, testing technology, and applying user-based approaches for improving the lives of older adults with dementia living in the community and LTHFs. The specific objectives are identifying LL activities linked to older adults with dementia; describing the fields of action of LLs dedicated to older adults with dementia and the types of research they conduct, investigating the technologies cocreated in LLs to improve the independence and quality of life of older adults with dementia, considering the impact of such solutions with regard to how effectively they reduce burdens on informal and formal caregivers, and addressing how LLs involve various stakeholders in identifying needs and finding solutions for older adults with dementia so that they can live more independently and with a better quality of life.

## Methods

### Overview

This scoping review was based on the guidelines published by Tricco et al [35]. The research protocol for this scoping review has been documented elsewhere [34]. Studies were included if they provided a description of the cocreation process; research methodology or design; the stakeholders involved; the impact or effects on independence or quality of life; or the impact or effects on health status, as defined by the authors. Studies were included if they were conducted within LLs or by researchers and managers (eg, health care professionals, ICT experts, and engineers) attached to an LL and working with older adults with dementia living in the community or LTHFs.

### Outcomes

The primary outcomes were information on the nature, number, and assessment of studies conducted with older adults with dementia performed by or in collaboration with LLs. Secondary outcomes were information on the documentation produced by different types of LLs, their objectives, the location of their interventions, and the types and methods of cocreation used for developing technologies and services for older adults and other stakeholders.

### Search Strategy

The search was conducted by a medical librarian (JRA) in March 2020. Six bibliographic databases—were searched—Embase.com, MEDLINE Ovid, PubMed (not MEDLINE[sb]), CINAHL EBSCO, APA PsycINFO Ovid, and the Web of Science Core Collection—with no language or date restrictions. The detailed search strategies are available in Multimedia Appendix 1. Additional searches were conducted in Google Scholar in French and English, and the *Journal of Engineering and Technology Management* (ISSN 0923-4748), *Technology Innovation Management Review* (ISSN 1927-0321), and the *Journal for Virtual Organization and Networks* (ISSN 1741-5225) were manually searched. A forward citation search based on key articles was conducted in the Web of Science Core Collection and Google Scholar in January 2021. Two members of the research team (HV and EP) performed reference screening and reviewed the bibliographies of the selected studies.

### Study Screening, Data Collection Process, and Data Items

Two reviewers (HV and EP) independently reviewed the abstracts and full text papers. In cases of disagreement, a consensus was reached through discussions and consultations with the coauthors. The research team developed Microsoft Excel spreadsheets to tabulate data on the studies and interventions and on their study quality assessments. The following information was extracted from each relevant study included and put into an appropriate usable form: (1) study authors, year of publication, and country where the study was conducted; (2) study characteristics (including research questions, study setting and design, sample size, instruments used, duration of follow-up, and stakeholders involved); (3) participants’ characteristics (including age, sex, health status, and place of living); and (4) types of outcome measures [36].

### Methodological Quality

The quality assessment of the selected papers was conducted using the Joanna Briggs Institute’s critical appraisal tools for quantitative, qualitative, and mixed methods studies [37]. Studies were not excluded based on their quality assessment as we wanted to provide an overview of the available information and its extent.

### Data Synthesis

The results are summarized using descriptive narrative synthesis. All data on LLs were integrated into a table.

## Results

### Search Strategy

**Figure 1:**
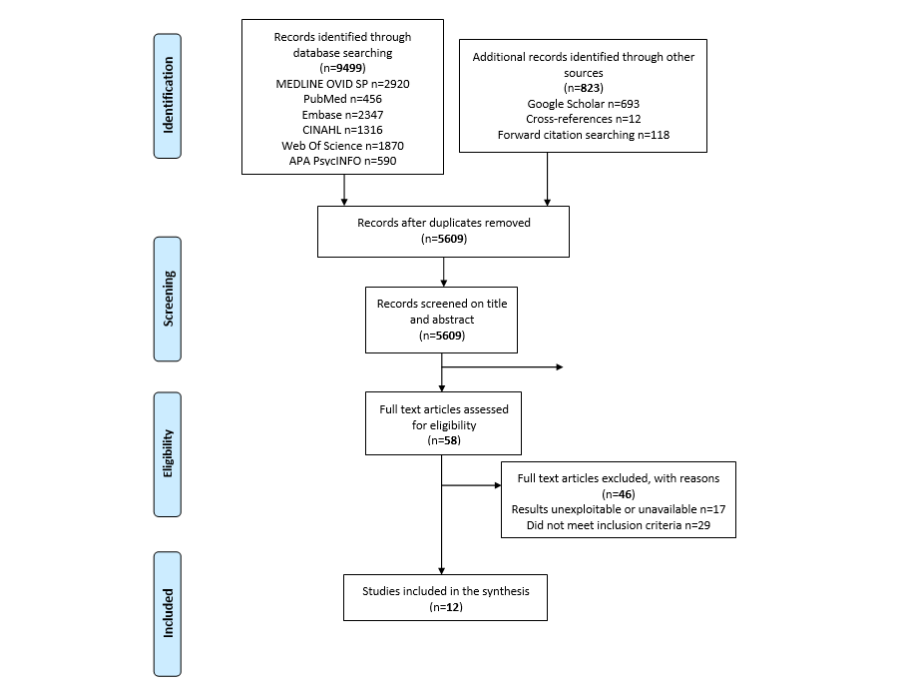
Flow diagram summarizing the results of the search strategy based on the PRISMA (Preferred Reporting Items for Systematic Reviews and Meta-analyses) recommendations [38].

Our strategy of searching bibliographic databases retrieved 5609 articles after eliminating duplicates. On the basis of their titles and abstracts, 58 articles were retained as potentially eligible, and their entire texts were evaluated. A total of 12 studies satisfied the selection criteria and were included (Figure 1 [38]).

### Characteristics of Studies and Participants

The 12 included studies were conducted in Canada, France, and the Netherlands and were published between 2009 and 2020 (Tables 1-3; Multimedia Appendix 2 [39-50]). These included 4 case studies, 3 mixed methods studies, 3 qualitative studies, 1 quasi-experimental study, and 1 quantitative, iterative pilot usability study. All these studies presented an innovative product to assist older adults with cognitive disorders or dementia.

**Table 1:** Characteristics of the included study from the Médéric Alzheimer Foundation living lab in France.

Study	Product	Design	Setting and sample	Method	Results	Quality of life; independence; caregivers
Charras et al [44]	“Dance intervention”: a modern and classical dance teacher with a nursing background led a 50-minute dance intervention	Mixed methods study	Day-care center: n=23 older adults with Alzheimer disease (12 women and 11 men) Mean age 83.47 (SD 5.4) years	Quantitative data: Get-Up and Go test; Stop Walking when Talking test; one-leg balance test Balance Confidence Scale Quality of Life in Alzheimer Disease Well-being: participant’s feedback Qualitative data: verbalization, behaviors, and attitudes noted in a logbook	Immediately enhanced well-being of 86% of participants, shorter execution time in the Get-Up and Go test for 66% of participants, variations in participants’ social behaviors	Strengthened physical condition and well-being

**Table 2:** Characteristics of the included studies from the Innovate Dementia living labs in four regions of northwest Europe (Belgium, Germany, the Netherlands, and the United Kingdom).

Study	Product	Design	Setting and sample	Method	Results	Quality of life; independence; caregivers
Brankaert and den Ouden [45]	“Qwiek Play”: a media system that creates an ambient experience through visual projection and sounds	Case study	Long-term care units: Study 1: n=14 residents with advanced dementia; n=6 care staff in care home for 29 days Study 2: n=11 residents with advanced dementia; n=4 care staff in a care home for 33 days Study 3: n=28 residents with moderate dementia; n=3 care staff in day-care center for 35 days	Sequence of activities: System and research method explained to staff Staff members invited to use and experiment with the system during the study period and record their experience on an evaluation form After the study period, additional insights collected during the focus group discussions with care professionals	The system had a considerable potential for people with dementia: could reduce need for medication and could help with better sleep. The system increased the efficiency of care provision by giving nurses more time to engage in care practices.	Reduction of agitation and aggression Improvement of quality of care and reduction of burden on formal caregivers
Suijkerbuijk et al [46]	“Aangenaam”: personal evaluation game with question cards “Vitaallicht”: dynamic light system that positively influences the sleep and wake cycle	Qualitative study	Community: 12 households; 5 women and 7 men with dementia, and their partners Mean age 74.92 years (SD 6.17; range 66-87 years)	Aangenaam: two methods—8 households received personal evaluation game and 4 received a tablet-based questionnaire Semistructured reflection and questionnaire administered at start and after first and second week Participants used Vitaallicht for 3 weeks Questionnaire to assess sleep quality	Aangenaam results: helped to capture first-person perspectives; a more appropriate research tool for people with dementia; enabled detailed capture of participants’ daily lives due to the diverse types of input Vitaallicht results: slight increase of mean subjective sleep quality over 2 weeks	Improved sleep quality
Brankaert et al [47]	“GoLivePhone”: smartphone app for communication, personal navigation, and sending out an emergency signal to caregivers	Qualitative study	Community: n=10 older adults with dementia and their informal caregivers	3 home visits over 3 weeks GPS data: activity levels data to see how often the phone was used Questionnaire: evaluate experiences and perspectives of participants or caregivers Reflection sessions: on technology and study	Device data: phones were used very irregularly Questionnaire: half of participants had positive experiences Reflection session: most comments pertained to positive experiences; some difficulties related to technological errors	Strengthened personal safety and independent walking Helped to reassure informal caregivers
Brankaert and den Ouden, [48]	“PhysiCAL”: activity reminder calendar to improve day’s structure and independence	Case study	Community: n=4 couples (1 older adult with dementia and 1 informal caregiver)	For 1 week, 4 couples used PhysiCAL at home. Perceptions collected through interviews	All the older adults with dementia said they did not need the device, but half of the caregivers noted that it was valuable	Stimulation of independence Reduction of informal caregiver’s burden

**Table 3:** Characteristics of the included studies from DOMUS (Laboratoire de Domotique et informatique Mobile à l’Université de Sherbrooke) in Canada.

Study	Product	Design	Setting and sample	Method	Results	Quality of life; independence; caregivers
Imbeault et al [49]	“AP@LZ”: electronic organizer to support day-to-day activities and help people to compensate for memory problems	Quasi-experimental study	Community: 3 older adults with Alzheimer disease (aged 71 years, 58 years, and 78 years)	Measures at 0, 3, 6, and 12 months Impact on daily living: Multifactorial Memory Questionnaire and Prospective and Retrospective Memory Questionnaire; personalized observation journals Impact on psychological components: Geriatric Depression Scale and Caregiver Burden Inventory	Postintervention: participants continued to use the system for managing appointments and making phone calls. Depressive symptoms did not significantly change in intensity. Decrease in perceived caregiver burden observed for one participant	Stimulated independence Reduced informal caregiver’s burden
Bier et al [50]	“SemAssist”: a computer program to assist people with semantic aphasia perform different steps of an activity	Case study	Community: one 68-year-old woman with semantic dementia	Therapy comprised preparing a target recipe. The participant was asked to generate semantic attributes of ingredients found in one target, one control, and two no-therapy recipes. The study took place over a 1-year period	Generated semantic attributes of ingredients pertaining to the target, and control recipes increased significantly (*P*<.001) as compared with no-therapy recipes (*P*=.79). The proportion of cooked meals was increased significantly (*P*=.02)	Stimulated independence

Of the 147 older adults who participated in these studies, 28 (19%) presented with mild cognitive impairment (MCI), 39 (26.5%) had Alzheimer disease, 12 (8.2%) presented with early-stage dementia, 42 (28.6%) presented with moderate dementia, 25 (17%) presented with advanced dementia, and 1 (0.7%) presented with semantic dementia. The participants’ ages ranged from 66 to 96 years. All studies included men and women. There were eight studies that were conducted in community settings, three in LTHFs, and one in a day-care center. Finally, 27 family caregivers—the partners of older adults affected by cognitive disorders or dementia—and 13 health care professionals were also included in these studies.

### Methodological Quality of the Studies

Measured using the Joanna Briggs Institute’s critical appraisal tools, the overall methodological quality of the studies included in this review was poor to moderate [37]. Only the study by Bier et al [50] was evaluated as having high methodological quality (Table 4).

**Table 4:** Critical appraisal results for included studies using the Joanna Briggs Institute’s Critical Appraisal Checklists.

Study design				Appraisal questions																		
				Question 1		Question 2		Question 3		Question 4		Question 5		Question 6		Question 7		Question 8		Question 9		Question 10
**Mixed methods study**																						
	**Quantitative analysis^a^**																					
		Charras et al [44]	Yes		Yes		Yes		Yes		No		No		Yes		Yes		—^b^		—	
		Wu et al [39]	Unclear		Yes		Yes		Yes		No		No		Yes		Yes		—		—	
		de Sant’Anna et al [43]	Yes		Unclear		Yes		Yes		No		No		Yes		Yes		—		—	
	**Qualitative analysis^c^**																					
		Charras et al [44]	N/A^d^		Yes		Yes		Yes		Yes		No		No		No		Yes		Unclear	
		Wu et al [39]	N/A		Yes		Yes		Yes		Yes		No		No		Yes		Yes		Yes	
		de Sant’Anna et al [43]	N/A		Unclear		Unclear		Unclear		No		No		No		No		Unclear		Yes	
**Quasi-experimental study^e^**																						
	Imbeault et al [49]			Yes		No		Yes		No		Yes		Yes		Yes		Yes		Yes		—
**Quantitative study^f^**																						
	Boulay et al [42]			No		Yes		Yes		Unclear		No		No		Unclear		Unclear		—		—
**Qualitative study^g^**																						
	Suijkerbuijk et al [46]			N/A		Yes		Yes		Yes		Yes		No		No		Yes		No		Unclear
	Brankaert et al [47]			N/A		Yes		Yes		Yes		Yes		No		No		Yes		No		Unclear
	Wu et al [40]			N/A		Yes		Yes		Unclear		Unclear		No		No		No		Yes		Unclear
**Case study^h^**																						
	Brankaert and den Ouden [45]			Unclear		No		Unclear		Yes		Yes		N/A		Yes		Yes		—		—
	Brankaert and den Ouden [48]			No		No		No		Unclear		Yes		N/A		Yes		Yes		—		—
	Bier et al [50]			Yes		Yes		Yes		Yes		Yes		Yes		Yes		Yes		—		—
	Faucounau et al [41]			Yes		No		Yes		Unclear		Yes		N/A		Yes		Yes		—		—

^a^Joanna Briggs Institute’s Critical Appraisal Checklist for analytical cross-sectional studies [37].

^b^No appraisal question.

^c^Joanna Briggs Institute’s Critical Appraisal Checklist for qualitative research [37].

^d^N/A: not applicable.

^e^Joanna Briggs Institute’s Critical Appraisal Checklist for quasi-experimental studies [37].

^f^Joanna Briggs Institute’s Critical Appraisal Checklist for analytical cross-sectional studies [37].

^g^Joanna Briggs Institute’s Critical Appraisal Checklist for qualitative research [37].

^h^Joanna Briggs Institute’s Critical Appraisal Checklist for case reports [37].

### Description of the Included Studies

The studies included in our evaluation were conducted in four LLs playing pivotal roles in developing innovations aimed at older adults with MCI or dementia and at their family or professional caregivers. These projects all aimed to contribute to optimizing the health, quality of life, independence, home care, and safety of older adults with MCI or dementia and to support their family and professional caregivers or reduce their burdens (Tables 1-3; Multimedia Appendix 2).

The LUSAGE (Laboratoire d’analyse des Usages en Gerontechnologies) LL, affiliated with the Geriatrics Department of the Broca Hospital and Paris Descartes University in France, specializes in the design, development, and supply of products and services providing assistive technologies to older adults with cognitive impairment (eg, MCI, Alzheimer disease, and related dementias) as well as their family and professional caregivers (Multimedia Appendix 2) [51]. LUSAGE is a partner laboratory of the National Expert Center in Cognitive Stimulation, launched by the National Solidarity Fund for Autonomy, whose main objective is to promote the development and use of innovative cognitive interventions. The European Network of Living Labs (ENoLL) certified LUSAGE in 2012, which has a flexible architectural configuration that can be adapted to conduct in situ observations (eg, home-like settings) according to each project’s requirements. LUSAGE develops solutions in assistive technologies in collaboration with their primary end users and stakeholders, which represents a multidisciplinary team comprising specialists from numerous fields such as researchers in geriatrics, technology, cognitive sciences, public health, law, and ethics, in addition to psychologists, physicians, engineers, designers, sociologists, and health economists. LUSAGE’s primary end users are older adults with cognitive disorders (recruited from the Broca Memory Clinic, Centers for Local Information and Coordination, and local Alzheimer associations), healthy older individuals, their families, and their informal and professional caregivers. These end users are involved in every stage of the product development cycle (eg, needs gathering, usability testing, monitoring studies, evaluation of technology acceptance, and ethical issues) [51].

One of LUSAGE’s primary activities is to test the utility and acceptability of personal assistance robots in older adults’ everyday lives (Multimedia Appendix 2). In 2014, Wu et al [39] simulated participants’ homes and compared how using the *Kompai* robot (Kompaï Robotics, Robosoft) to complete daily tasks affected the lives of 6 older adults with MCI and 5 others in good cognitive health. Participants with MCI were able to use *Kompai* just as well as those with good cognitive health. However, despite the robot’s positive attributes, such as its ease of use and playful dimension, participants reported that they had no intention of using a personal assistance robot in their daily life as they had negative perceptions about this type of device, associated with negative representations of dependence linked to aging [39]. With the aim of improving the acceptability of personal assistance robots for the homes of older adults with MCI, LUSAGE subsequently ran the Robadom project [40]. The objective of Robadom was to define an ideal robot, in appearance and functionality, that would meet the expectations of older adults with MCI. The most appreciated functions were cognitive stimulation, object finding, and diary reminders about upcoming events, such as the need to take medication or go to an appointment. Most of the participants had negative perceptions of robots with human characteristics and preferred short robots with stylized, rounded, discrete, and yet familiar shapes [40].

Another innovation developed by LUSAGE was using GPS to improve the independence, quality of life, and safety of home-dwelling older adults with dementia and to help their family caregivers [41]. A mobile telephone attached to the older adult’s belt provided standard telephone functionalities, but it also transmitted geolocation data to the family caregiver by SMS text messages and could send numerous alarms. Faucounau et al [41] tested this device for a month in the daily life of an 84-year-old man with Alzheimer disease and his wife. The couple’s general impressions were that the device was too bulky, sometimes gave imprecise location coordinates, and had a poor battery life [41].

Finally, LUSAGE has also been used to develop and test innovations in LTHF settings [42,43]. In 2011, Boulay et al [42] tested their MINWii device with 7 older adults with Alzheimer disease institutionalized in a nursing home. MINWii mixes music therapy and cognitive stimulation by allowing players to improvise or play songs of their choice by pointing at a virtual keyboard with a Wii remote control. Numerous benefits of the MINWii, such as positive stimulation of cognitive function, participants’ ability to reminisce, and easier interactions with the care team, have been reported [42]. Sant’Anna et al [43] evaluated the impact of using a seal-shaped robot named Paro on the capacity to communicate and the behaviors of 5 nursing home residents with severe Alzheimer disease. Quantitative results indicated that using Paro led to a significant reduction in *disturbed behaviors* (*P*=.04), especially anxiety, aggressivity, irritability, and sleep disorders. A positive change in communication skills and abilities was also noted in 4 of the 5 patients. Thus, Paro seemed to be an excellent facilitator of communication for older adults with Alzheimer disease, inciting verbal and tactile communication as well as the expression and transfer of feelings by voice and touch [43].

A second French LL working on projects aimed at older adults with dementia was set up in Versailles in 2017 by the Médéric Alzheimer Foundation (Table 1). It focuses on developing and evaluating innovative responses in this field to improve the integration and quality of life of older adults with Alzheimer disease or related illnesses [52]. This LL collaborates in a coparticipative manner with older adults and their family caregivers, treating them as both actors and experts in their disease. It also works with health care professionals, researchers, and entrepreneurs. The central focus of the Foundation’s LL is evaluating the impact of various psychosocial interventions, such as cognitive stimulation, art therapy, music therapy, or reminiscence, on the quality of life of older adults with Alzheimer disease.

In 2020, Charras et al [44] evaluated the impact of a dance therapy intervention on 23 older adults with Alzheimer disease who regularly attended a day-care center. The study’s results revealed that 86% of participants (*P*<.001) experienced a significant increase in well-being immediately after a dance session, and 66% of them (*P*=.04) also showed a tendency toward faster times in a balance test [44].

A third European grouping of LLs focuses on developing innovative solutions for older adults with dementia. The Innovate Dementia Project comprises ten partners in four regions of Northwestern Europe (Belgium, Germany, the Netherlands, and the United Kingdom), and they collaborate via more than 25 LLs to explore, develop, test, and evaluate innovative, sustainable solutions that consider the socioeconomic challenges linked to aging and dementia (Table 2) [53]. Their goal is to improve the quality of life and independence of older adults with dementia and to facilitate the support given to them by their close family caregivers. This project began in 2012 and became a member of the ENoLL network in 2014, concentrating on four issues: intelligent lighting systems, nutrition and physical exercise, living environments, and models of assistance. The Innovate Dementia Project allows end users (persons living with dementia and their family caregivers), whose role is central, to collaborate with different stakeholders (care professionals, businesses, academic and knowledge institutes, and local governments) to develop and test innovative products in real-life conditions, notably in the homes of older adults with dementia. To date, this project has involved 500 end users, more than 200 health care professionals, and more than 25 business partners, and these partnerships have allowed them to bring more than 15 innovative solutions to the market.

In 2013, Brankaert and den Ouden [48] presented the results of the first product to be tested at the Eindhoven LL: PhysiCAL, a personal activity reminder calendar that promotes older adults’ independence. All of the participating older adults with dementia stated that they did not need such a device, whereas 3 of the 4 family caregivers thought that it had helped [48]. In 2014, Brankaert et al [47] trialed a second product, GoLivePhon*e*, in the homes of 10 older adults with dementia and their family caregivers. The phone had three main functions: communicating with other people, providing support when out in the community via a personal navigation system, and sending an emergency signal to a family caregiver. Family caregivers were able to monitor and consult their partners’ smartphones via a web-based app, *GoLiveAssist*. Although the app was used irregularly and several technical errors occurred during the trial period, slightly more than half of the participants reported having had a very positive experience and that the device had been helpful. Family caregivers reported that they were reassured by the device as it improved their partner’s support and safety [47]. A 2016 study by Suijkerbuijk et al [46], conducted in the homes of 12 couples where one of the pairs had dementia, managed to test two innovative products at the same time: the Aangenaam personal evaluation game and the Vitaallicht dynamic light system. The Aangenaam system enables informal data collection on the daily lives of older adults with dementia, and as it takes the form of a game, it has a minimal risk of disturbing their ADLs. The older adult picks a card from a deck and can answer the question in different ways, by writing in a notebook, by answering orally to make an audio recording, or by taking photographs with the camera provided. The questions explored four categories of data: experiences linked to the ADLs, the participant’s social and physical context, their personal objectives and significant life events, and a category adaptable to the product or device being tested. As compared with using a questionnaire on a tablet computer, the findings revealed that the Aangenaam system was better suited and more appreciated by participants; however, thanks to the different potential means of response, it also allowed the researchers to gather more details about their daily lives [46]. The Vitaallicht product, for its part, is a dynamic lighting system that uses blue light to positively influence sleep-wake cycles by suppressing melatonin production during the day. After only 2 weeks of use, this system induced a subjective increase in the quality of the participants’ sleep [46]. One of the latest products developed by the Innovate Dementia Project is the Qwiek Play media system, which creates a calming ambient experience in a room by projecting images and sound (a walk through the woods, looking up at a starry sky, visiting a farm, or viewing a custom slideshow of family photos accompanied by music). This product was used in 2017 by Brankaert and den Ouden [45], with 25 patients with severe dementia living in nursing homes and 28 older adults with moderate dementia attending a day-care center. The impressions of the 13 health care professionals were also explored. The results reported very positive perceptions about the product, mentioning its potential for use in nonmedicated interventions to reduce stress and agitation in older adults with moderate to severe dementia, thus giving care staff more time to engage in their care practices [45].

Our scoping review identified a final LL aimed at helping older adults with dementia: DOMUS (Laboratoire de Domotique et informatique Mobile à l’Université de Sherbrooke) in Canada (Table 3) [54]. Set up in 2014, this LL represented the first project of its type in Canada, and it is equipped with a rich, multipurpose infrastructure for the design, implementation, and evaluation of different types of cognitive orthotics. The resulting set of orthotics support a wide variety of ADLs (eg, medication, meal preparation, or budgeting), fostering greater independence at home for people with cognitive impairments (Alzheimer disease, mental retardation, schizophrenia, or traumatic brain injury). DOMUS operates three variants of the LL concept: a smart apartment on its campus that is controlled by a home automation system enabling short-term studies in technology-rich simulated housing; an LL in an alternative housing unit for people with traumatic brain injury, enabling long-term ecological studies in a technology-rich real house; and the LL *at home* that can be installed in older adults’ places of residence (apartments and houses), enabling long-term ecological studies in a mobile, agile-technology environment. From the beginning of each project, developing cognitive orthoses involves implicating end users (older adults with cognitive disorders and people with traumatic brain injury) with other stakeholders (clinical researchers, engineers, health care professionals, gerontologists, occupational therapists, neuropsychologists, and researchers in ergonomics and design) to ensure that assistive technologies are focused on users and fully satisfy their needs [54].

In 2011, Bier et al [50] tested a cognitive assistance product named SemAssist with a 68-year-old woman living alone and who had semantic dementia. This device helps people with semantic aphasia in performing different stages of an activity. Findings showed that this therapy, involving following the same targeted recipe several times over a year, helped this woman reduce the number of errors she made while preparing that recipe. The intervention stimulated her memory function as food preparation developed new episodic memories surrounding the following recipes. Thanks to SemAssist, the participant’s self-confidence in being able to cook also grew, which encouraged her to do so more often. The proportion of meals that she cooked for herself increased significantly (*P*=.02) [50]. Finally, in 2018, Imbeault et al [49] tested the AP@LZ smartphone app in the homes of 3 older adults with Alzheimer disease. The goals were to optimize their independence in ADLs by compensating for their memory problems, further supporting family caregivers and alleviating their burdens. The AP@LZ works like a personal assistant or organizer and has five main functions, namely appointment reminders, a personal database, a medical database, a list of contacts, and a notepad for jotting down shopping lists. The 3 participants had different profiles with respect to age, cognitive status, and social status. Participant 1 was a 71-year-old married man diagnosed with Alzheimer disease 1 year earlier, who had language problems and both verbal and visual memory deficits. Participant 2 was a 58-year-old married man diagnosed with atypical Alzheimer disease 1 year earlier, dominated by dysexecutive syndrome and constructive and ideomotor apraxia. Participant 3 was a 78-year-old single woman living alone in sheltered housing and diagnosed with Alzheimer disease 1 year earlier, which mainly manifested a memory disorder. The findings underlined that all 3 participants, despite their different profiles, could use the app in their everyday lives. Indeed, they all continued to use it after the study ended as they found that the system helped them, and they especially appreciated the appointment reminder function. Using AP@LZ also reduced the burden on family caregivers. The authors concluded that the app might have long-term utility, despite Alzheimer disease being a progressive disease and that it could be used by people with different profiles and degrees of cognitive impairment [49].

## Discussion

### Principal Findings

This review aimed to identify publications examining all types of LL activities, exploring the needs and expectations of older adults with dementia and looking for solutions, whether they were living in the community or in LTHFs. We discovered 12 studies that met our inclusion criteria (quantitative, qualitative, or mixed methods) involving 147 older adults with MCI or dementia, 27 informal caregivers, and 13 formal caregivers. These studies originated from three European LLs and one Canadian LL playing key roles in research in this field. Their work has allowed the development, testing, and evaluation of a series of innovative products aimed not only at optimizing the health, quality of life, independence, home care, and safety of older adults with MCI or dementia but also at supporting formal and informal caregivers and reducing their levels of burden. Most of the studies in this scoping review reported promising findings, and the LL approach highlighted both positive and negative points in all the devices, products, and services, which will be open to improvements through future testing.

### Limitations

This scoping review has some limitations. Our literature search strategy may have omitted some studies as they did not meet all our inclusion criteria or as researchers failed to identify them in the study selection process. Some bias might have also been present in the reporting of findings by the investigators in the analysis of the selected studies. It is impossible to exclude some bias in the selection of studies as all the included studies had very limited sample sizes. Indeed, only one of the studies was evaluated as having a high methodological quality. The limited number of participants and the overrepresentation of European LLs means that generalizing these findings to a broader population or other countries should be done with great care. Finally, the limited number of recent studies revealed by this scoping review raises questions about whether any LL activities are ongoing and whether LLs are sustainable.

To the best of our knowledge, there are no previous, clear overviews of the research conducted by LLs with respect to older adults with cognitive impairment or dementia. Our scoping review has allowed us to understand the services, research, and clinical activities developed in different LL settings for older adults with dementia. Therefore, it provides valuable information to nurses, general practitioners, policy makers, and other stakeholders involved in LLs dedicated to older adults. Furthermore, the diversity of the research projects that we included managed to test the innovative solutions using a variety of methodologies.

### Comparison With Previous Work

LLs represent a promising approach for developing innovative solutions to the numerous challenges of an increasingly older population [3]. Indeed, it can offer an ideal, pragmatic framework for research involving a realistic, real-life setting, multiple stakeholder participation, multi-method approaches, and cocreation [55].

To the best of our knowledge, there are no best practices for design-driven LLs. The lack of consensus on the practices, methods, tools, and boundaries of LLs raises several obstacles to the adoption of this approach as well as creating confusion about the definition and components of an LL [56]. Thus, some research groups claim to be using an LL approach, although they really are not. In contrast, some research groups using LL approaches are not labeled as such. For example, the ENoLL label is so new that it has not yet been classified as an LL [15]. Furthermore, the complexity and diversity of what is going on within an LL can blur the boundaries among research, industry, and other economic market sectors [15]. Multifactorial difficulties in finding financing for LLs are another frequently reported problem (instability over the medium to long term, problems balancing representativity between stakeholders in decision-making, and investors’ different expectations with regard to returns on investment, and the absence of social capital). Managing intellectual property is also problematic because of the lack of a consensus model for doing this and the ad hoc nature of contractual dealings and agreements [15,56]. Finally, several difficulties have been reported concerning the sustainability of LLs [57]. Primarily because of the notable lack of sustainable financing or nondiversified financing (whether from private or public sources), it is common for LLs not to survive beyond the time needed to conduct their first financed research project [57]. Thus, it seems essential that to have sustainable LLs, they should be developed within solid, dynamic, long-term, strategic frameworks that continuously evaluate financing, new target audiences, and potential revenue streams. They should involve multiple stakeholders and have the capacity to evolve over time, moving from one innovation category to another [57].

With regard to projects aimed at older adults, numerous studies conducted in LLs aim to find solutions to the pressing problems facing older populations in general [26]. However, there are still few innovative tools, solutions, or technologies that are especially adapted for older adults with dementia [25]. It will be essential to promote more research and experiments in LLs aimed at populations with dementia as these approaches are promising and encourage the cocreation of innovative solutions to maintain or improve their health, quality of life, and independence [58,59]. Although integrating older adults with dementia into the LL process—from product design to evaluation—is also essential, it remains sporadic, unfortunately, because of the inherent difficulties of collaborating with individuals with an impaired cognitive function and the ethical issues that this raises [29]. LL approaches too often only include formal and informal caregivers when older adults are still capable of participating, and innovative solutions will never be optimally effective if they fail to fully meet their needs and expectations [29]. Several studies have reported that older adults with dementia would be happy to actively participate in the development processes seeking innovative solutions that would benefit them in the future. They are enthusiastic about the idea of contributing to these solutions by bringing their unique and precious experiential knowledge [60]. The LL approach represents an ideal research and experimental framework for older adults with dementia as studies that fail to include them as coparticipants will not be able to meet their real-world needs and reliably show the effects of innovative solutions on this population’s daily lives [29]. There are numerous strategies to ensure the voluntary participation of older adults with dementia and overcome the challenges of cognitive impairments and ethics, such as the concepts of fluctuating consent, process consent, or rolling consent. These strategies promote effective communication between all stakeholders involved so that the vulnerable person’s willingness to participate can be monitored continuously [61]. A complete LL approach must necessarily involve formal and informal caregivers as innovative solutions must meet the needs and expectations of end users and those who look after them [54]. The LL approach also requires the points of view, expertise, and collaborations of all the involved stakeholders (eg, students, academic institutions, private companies, health care organizations, and patient representative bodies) [62]. Given that most LLs focusing on older adults with dementia appear to be in Europe, this approach requires development on other continents [63]. We do not know of any best practices for design-driven LLs, and it may be necessary to develop guidelines on the LL approach to direct and support the establishment and sustainability of innovative solutions, and to facilitate relationships and engagement with stakeholders and end users [45].

### Conclusions

To the best of our knowledge, there are no clear views of the research conducted by LLs with respect to older adults with cognitive impairments or dementia. This scoping review enabled us to draw together the few but varied existing research findings and contributed to consolidating knowledge in this field. This allowed us to identify 4 LLs that play a central role in research testing and evaluating innovative products to optimize the health, quality of life, independence, home care, and safety of older adults with dementia, whether they live in their homes or in LTHFs. This research also supports and reduces the burden on formal and informal family caregivers. Furthermore, this scoping review could be used as a reference for anybody interested in using LLs with older adults with cognitive impairments or dementia. It provides valuable information to nurses, general practitioners, policy makers, and other stakeholders involved in LLs dedicated to older adults on the practices, methods, and tools that can be used with older adults with cognitive impairment or dementia. To date, very few studies using the LL approach have focused on older adult populations with dementia, notably because of the difficulties associated with their lower cognitive abilities and the ethical challenges this raises. By allowing older adults with dementia to experience cocreation within a well-defined environment and influence a potential product’s design, ease of use, or acceptability, the other stakeholders should be better able to address their needs and expectations. Therefore, it is essential that more LL experiments integrate both older adults with dementia, their formal and informal caregivers, and all other pertinent stakeholders. This will assist in the development of more appropriate, better adapted, sustainable, innovative interventions, services, and products to meet the growing societal challenges brought on by dementia.

## Appendix

Multimedia Appendix 1 Bibliographic database search strategies.

Multimedia Appendix 2 Characteristics of the included studies from the LUSAGE (Laboratoire d’analyse des Usages en Gerontechnologies) living lab in France.

## Abbreviations

ADL: activities of daily living
DOMUS: Laboratoire de Domotique et informatique Mobile à l’Université de Sherbrooke
ENoLL: European Network of Living Labs
ICT: information and communication technology
LL: living lab
LTHF: long-term health care facility
LUSAGE: Laboratoire d’analyse des Usages en Gerontechnologies
MCI: mild cognitive impairment
