# Supplementary material for: Using Living Labs to Explore Needs and Solutions for Older Adults With Dementia: Scoping Review
Source: JMIR Aging. 2021 Aug 19;4(3):e29031. doi: 10.2196/29031 (PMC8414306; doi:10.2196/29031)
Supplement: Multimedia Appendix 1 [file aging_v4i3e29031_app1.docx]

Using Living Labs To Explore Needs And Solutions For Older Adults With Dementia: A Scoping Review

**Multimedia Appendix 1: Bibliographic database search strategies**

Note: the research strategies were peer reviewed by another information specialist prior to execution.

**Embase.com**

Last Content Update 30 Mar 2020 08:23:02 GMT

March 30, 2020

2347 references found

('cognitive defect'/exp OR 'amnesia'/exp OR 'delirium'/de OR 'aged'/exp OR 'elderly care'/de OR 'geriatric care'/exp OR 'geriatric patient'/de OR 'geriatrics'/exp OR (dementia* OR "cognitive impair*" OR "cognitive defect" OR alzheimer OR amnesia OR "neurocognitive disorder*" OR "cognition disorder*" OR "traumatic psychose*" OR Korsakoff OR Huntington OR "Lewy Body" OR delirium OR elder* OR eldest OR geriatr* OR "old age*" OR (older NEXT/1 (patient* OR people OR subject* OR age* OR adult* OR man OR men OR woman OR women OR population* OR person*)) OR aging OR ageing OR senior* OR "late life"):ab,ti,kw) AND ('participatory research'/de OR ("living lab*" OR co-creat* OR cocreat* OR co-design* OR codesign* OR co-research* OR co-conception OR ((co-produc* OR co-develop*) AND (research* OR model* OR technolog*)) OR (participatory NEAR/4 (research OR design)) OR (iterative NEXT/3 design*) OR "designing technolog*" OR (innovation NEXT/3 communit*) OR "user innovation" OR "community pilot" OR "experimental lab*"):ab,ti,kw)

**Medline Ovid SP**

Ovid MEDLINE(R) and Epub Ahead of Print, In-Process & Other Non-Indexed Citations and Daily 1946 to March 23, 2020

March 25, 2020

2920 references found

(exp "Neurocognitive Disorders"/ OR exp aged/ OR "Geriatric Nursing"/ OR "Geriatrics"/ OR "Geriatric Psychiatry"/ OR (dementia* OR "cognitive impair*" OR "cognitive defect" OR alzheimer OR amnesia OR "neurocognitive disorder*" OR "cognition disorder*" OR "traumatic psychose*" OR Korsakoff OR Huntington OR "Lewy Body" OR delirium OR elder* OR eldest OR geriatr* OR "old age*" OR (older ADJ1 (patient* OR people OR subject* OR age* OR adult* OR man OR men OR woman OR women OR population* OR person*)) OR aging OR ageing OR senior* OR "late life").ab,ti,kf.) AND (exp "Community-Based Participatory Research"/ OR ("living lab*" OR co-creat* OR cocreat* OR co-design* OR codesign* OR co-research* OR co-conception OR ((co-produc* OR co-develop*) AND (research* OR model* OR technolog*)) OR (participatory ADJ3 (research OR design)) OR (iterative ADJ3 design*) OR "designing technolog*" OR (innovation ADJ3 communit*) OR "user innovation" OR "community pilot" OR "experimental lab*").ab,ti,kf.)

**PubMed**

March 30, 2020

456 references found

Limit : NOT medline[sb]

(dementia*[tiab] OR "cognitive impair*"[tiab] OR "cognitive defect"[tiab] OR alzheimer[tiab] OR amnesia[tiab] OR "neurocognitive disorder*"[tiab] OR "cognition disorder*"[tiab] OR "traumatic psychose*"[tiab] OR Korsakoff[tiab] OR Huntington[tiab] OR "Lewy Body"[tiab] OR delirium[tiab] OR elder*[tiab] OR eldest[tiab] OR geriatr*[tiab] OR "old age*"[tiab] OR (older[tiab] AND (patient*[tiab] OR people[tiab] OR subject*[tiab] OR age*[tiab] OR adult*[tiab] OR man[tiab] OR men[tiab] OR woman[tiab] OR women[tiab] OR population*[tiab] OR person*[tiab)) OR aging[tiab] OR ageing[tiab] OR senior*[tiab] OR "late life"[tiab) AND ("living lab*"[tiab] OR co-creat*[tiab] OR cocreat*[tiab] OR co-design*[tiab] OR codesign*[tiab] OR co-research*[tiab] OR co-conception[tiab] OR ((co-produc*[tiab] OR co-develop*[tiab) AND (research*[tiab] OR model*[tiab] OR technolog*[tiab)) OR (participatory[tiab] AND (research[tiab] OR design[tiab)) OR (iterative[tiab] AND design*[tiab) OR "designing technolog*"[tiab] OR (innovation[tiab] AND communit*[tiab) OR "user innovation"[tiab] OR "community pilot"[tiab] OR "experimental lab*"[tiab) NOT medline[sb]

**CINAHL EBSCO**

March 30, 2020

1316 references found

(MH "Delirium, Dementia, Amnestic, Cognitive Disorders+" OR MH "Alzheimer's Disease" OR MH "Aged+" OR MH "Gerontologic Care" OR MH "Gerontologic Nursing+" OR MH "Geriatrics" OR MH "Geriatric Psychiatry" OR TI (dementia* OR "cognitive impair*" OR "cognitive defect" OR alzheimer OR amnesia OR "neurocognitive disorder*" OR "cognition disorder*" OR "traumatic psychose*" OR Korsakoff OR Huntington OR "Lewy Body" OR delirium OR elder* OR eldest OR geriatr* OR "old age*" OR (older W1 (patient* OR people OR subject* OR age* OR adult* OR man OR men OR woman OR women OR population* OR person*)) OR aging OR ageing OR senior* OR "late life") OR AB (dementia* OR "cognitive impair*" OR "cognitive defect" OR alzheimer OR amnesia OR "neurocognitive disorder*" OR "cognition disorder*" OR "traumatic psychose*" OR Korsakoff OR Huntington OR "Lewy Body" OR delirium OR elder* OR eldest OR geriatr* OR "old age*" OR (older W1 (patient* OR people OR subject* OR age* OR adult* OR man OR men OR woman OR women OR population* OR person*)) OR aging OR ageing OR senior* OR "late life")) AND (TI ("living lab*" OR co-creat* OR cocreat* OR co-design* OR codesign* OR co-research* OR co-conception OR ((co-produc* OR co-develop*) AND (research* OR model* OR technolog*)) OR (participatory N4 (research OR design)) OR (iterative N4 design*) OR "designing technolog*" OR (innovation W4 communit*) OR "user innovation" OR "community pilot" OR "experimental lab*") OR AB ("living lab*" OR co-creat* OR cocreat* OR co-design* OR codesign* OR co-research* OR co-conception OR ((co-produc* OR co-develop*) AND (research* OR model* OR technolog*)) OR (participatory N4 (research OR design)) OR (iterative N4 design) OR "designing technolog*" OR (innovation W4 communit*) OR "user innovation" OR "community pilot" OR "experimental lab*"))

**APA PsycINFO OVID SP**

APA PsycInfo 1806 to March Week 4 2020

March 30, 2020

590 references found

(neurocognitive disorders/ OR exp dementia/ OR alzheimer's disease/ OR cognitive impairment/ OR exp Amnesia/ OR delirium/ OR geriatric patients/ OR exp geriatrics/ OR elder care/ OR exp aging/ OR (dementia* OR "cognitive impair*" OR "cognitive defect" OR alzheimer OR amnesia OR "neurocognitive disorder*" OR "cognition disorder*" OR "traumatic psychose*" OR Korsakoff OR Huntington OR "Lewy Body" OR delirium OR elder* OR eldest OR geriatr* OR "old age*" OR (older ADJ1 (patient* OR people OR subject* OR age* OR adult* OR man OR men OR woman OR women OR population* OR person*)) OR aging OR ageing OR senior* OR "late life").ab,ti.) AND ("living lab*" OR co-creat* OR cocreat* OR co-design* OR codesign* OR co-research* OR co-conception OR ((co-produc* OR co-develop*) AND (research* OR model* OR technolog*)) OR (participatory ADJ3 (research OR design)) OR (iterative ADJ3 design*) OR "designing technolog*" OR (innovation ADJ3 communit*) OR "user innovation" OR "community pilot" OR "experimental lab*").ab,ti.

**Web Of Science – Core Collection**

March 30, 2020

1870 references found

TS=(dementia* OR "cognitive impair*" OR "cognitive defect" OR "alzheimer" OR "amnesia" OR "neurocognitive disorder*" OR "cognition disorder*" OR "traumatic psychose*" OR "Korsakoff" OR "Huntington" OR "Lewy Body" OR "delirium" OR elder* OR "eldest" OR geriatr* OR "old age*" OR ("older" NEAR/1 (patient* OR "people" OR subject* OR age* OR adult* OR "man" OR "men" OR "woman" OR "women" OR population* OR person*)) OR "aging" OR "ageing" OR senior* OR "late life") AND TS=("living lab*" OR co-creat* OR cocreat* OR co-design* OR codesign* OR co-research* OR "co-conception" OR ((co-produc* OR co-develop*) AND (research* OR model* OR technolog*)) OR ("participatory" NEAR/4 ("research" OR "design")) OR ("iterative" NEAR/4 design*) OR "designing technolog*" OR ("innovation" NEAR/4 communit*) OR "user innovation" OR "community pilot" OR "experimental lab*")

**Google Scholar**

March 24, 2020

Search options

- Since 2016
- Do not include patents
- Do not include citations

dementia| "living lab"|"living labs"

200 first references consulted

démence "living lab"|"living labs"

19 references found
