# Supplementary material for: Using Living Labs to Explore Needs and Solutions for Older Adults With Dementia: Scoping Review
Source: JMIR Aging. 2021 Aug 19;4(3):e29031. doi: 10.2196/29031 (PMC8414306; doi:10.2196/29031)
Supplement: Multimedia Appendix 2 [file aging_v4i3e29031_app2.docx]

**Multimedia Appendix 2:** Characteristics of the included studies from the Laboratoire d'analyse des USAges en GErontechnologies living lab in France.

| Study | Product | Design | Setting and sample | Method | Results | Quality of life; independence; caregivers |
| --- | --- | --- | --- | --- | --- | --- |
| Wu et al [1] | “Kompaï”: a robot with a tablet PC that remembers appointments, manages shopping lists, plays music, and has a videoconferencing system. | Mixed methods study | Community: n=6 older adults with MCI^a^ and n=5 cognitively intact older adults; 9 women and 2 men; mean age 79.3 years (range 76-85 years) | - Participants interacted with the robot for 1 hour once a week for 4 weeks - Robot acceptance questionnaire - Semistructured interviews - Usability performance measures - Focus group | - All subjects able to use the robot - Low scores: intention to use, perceived usefulness, and attitudes toward robots - High scores: ease of use, social influence, perceived enjoyment, and anxiety - Barriers: uneasiness with technology and feeling of stigmatization | - Contributed to the quality of life - Stimulated independence |
| Wu et al [2] | “Robadom project”: goal to design a robot with emotions and language able to assist home-dwelling older adults with MCI. | Three qualitative studies and one mixed methods study | Community:   - First study: n=15 older adults with MCI - Second study: n=8 healthy older adults and n=7 with MCI - Third study: n=23 older adults and n=20 young subjects - Fourth study: n=19 older adults | - First study: semistructured interviews exploring needs and perceptions about the robot - Second study: focus group to define the robot’s ideal appearance - Third study: evaluate the perceptions of robot’s expressivity - Fourth study: compare the effects of 3 devices (a laptop computer, a robot, and an avatar) | - First study: cognitive stimulation, object searching, and reminders were appreciated - Second study: reticence toward robots with human characteristics - Third study: expressions best interpreted via colors and ear positioning - Fourth study: preference went to the laptop, then the robot, and finally the avatar | Contributed to physical, social, and psychological well-being and maintenance of quality of life |
| de Sant’Anna et al [3] | “Paro seal robot”: robot with sensors sensitive to light, touch, sound, and some sentences; expressions via flipper, neck, and eyelid movements | Mixed methods study | Long-term health care facilities: n=5 older adults with severe Alzheimer disease; age range 66-96 years | Eight individual sessions for 20 minutes over 4 weeks. Quantitative data:   - NeuroPsychiatric Inventory - Apathy Inventory - Cornell Scale for Depression in Dementia | Quantitative results: significant decrease (*P*=.04) in anxiety, aggressivity, irritability, and sleep quality. Impact on the expression of feelings, verbal and nonverbal exchanges, and patient’s search for intimacy and contact | Communication vector that improved the expression of emotions and quality of life |
| Boulay et al [4] | “MINWii” video game that plays songs by pointing at a keyboard | Pilot usability study | Long-term health care facilities: n=7 older adults with Alzheimer disease; 4 women and 3 men; mean age 88.5 years (range 77-94 years) | Testing sessions once a week for 10-20 minutes per patient. All sessions were videorecorded. | - Positive stimulation of cognitive abilities - Positive interaction with caregivers - Powerful reminiscence - Good satisfaction | Active Music Therapy treatment could improve the patients’ quality of life. |
| Faucounau et al [5] | “Global Positioning System”: with geolocation alarms via SMS text messages, voluntary alarm, alarms when going beyond the preset safety zone, detecting long inactivity, and falls | Case study | Community: n=1 older adult with Alzheimer disease (84 years) and his spouse (68 years) | Use of the device for 1 month | - Patient’s impressions pretest: removable system as desired, reassurance for his wife; posttest: device too voluminous and ugly - Caregiver’s impressions pretest: autonomous management of husband's wanderings; posttest: device too voluminous, malfunctions and usage difficulties, imprecise coordinates, and low battery autonomy | - Strengthened personal safety - Improved independent walking - Helped to reassure informal caregivers |

^a^MCI: mild cognitive impairment.

1. Wu YH, Wrobel J, Cornuet M, Kerherve H, Damnee S, Rigaud AS. Acceptance of an assistive robot in older adults: a mixed-method study of human-robot interaction over a 1-month period in the Living Lab setting. Clin Interv Aging. 2014;9:801-11. PMID: 24855349. doi: 10.2147/CIA.S56435.

2. Wu Y-H, Wrobel J, Cristancho-Lacroix V, Kerhervé H, Chetouani M. Le projet Robadom : conception d’un robot d’assistance pour les personnes âgées. Revue de Gériatrie, 2013. 2014;Tome 38 (5), pp.349-353.

3. de Sant’Anna M, Morat B, Rigaud AS. Interest of the Paro therapeutic robot in the management of institutionalised patients with severe Alzheimer’s disease. NPG Neurologie - Psychiatrie - Gériatrie. 2012;Volume 12, Issue 67, February 2012, Pages 43-48. doi: 10.1016/j.npg.2011.10.002.

4. Boulay M, Benveniste S, Boespflug S, Jouvelot P, Rigaud AS. A pilot usability study of MINWii, a music therapy game for demented patients. Technol Health Care. 2011;19(4):233-46. PMID: 21849735. doi: 10.3233/THC-2011-0628.

5. Faucounau V, Riguet M, Orvoen G, Lacombe A, Rialle V, Extra J, et al. Electronic tracking system and wandering in Alzheimer's disease: a case study. Ann Phys Rehabil Med. 2009 Sep-Oct;52(7-8):579-87. PMID: 19744906. doi: 10.1016/j.rehab.2009.07.034.
